# Associations of step accelerations and cardiometabolic risk markers in early adulthood

**DOI:** 10.1093/eurpub/ckae199

**Published:** 2024-12-04

**Authors:** Ville Stenbäck, Inka Lehtonen, Juhani Leppäluoto, Dominique Gagnon, Marjo-Riitta Järvelin, Mikko Tulppo, Karl-Heinz Herzig

**Affiliations:** Research Unit of Biomedicine and Internal Medicine, Faculty of Medicine, University of Oulu, Oulu, Finland; Research Unit of Biomedicine and Internal Medicine, Faculty of Medicine, University of Oulu, Oulu, Finland; Research Unit of Biomedicine and Internal Medicine, Faculty of Medicine, University of Oulu, Oulu, Finland; Faculty of Sports and Health Sciences, University of Jyväskylä, Jyväskylä, Finland; Helsinki Sports and Exercise Medicine Clinic (HULA), Foundation for Sports and Exercise Medicine (HULA), Helsinki, Finland; School of Kinesiology and Health Sciences, Laurentian University, Sudbury, Canada; Center for Life Course Health Research, Faculty of Medicine, University of Oulu, Oulu, Finland; Department of Epidemiology and Biostatistics, School of Public Health, Imperial College London, London, United Kingdom; Biocenter Oulu, University of Oulu, Oulu, Finland; Unit of Primary Care, Oulu University Hospital, Oulu, Finland; Department of Life Sciences, College of Health and Life Sciences, Brunel University London, Uxbridge, United Kingdom; Research Unit of Biomedicine and Internal Medicine, Faculty of Medicine, University of Oulu, Oulu, Finland; Research Unit of Biomedicine and Internal Medicine, Faculty of Medicine, University of Oulu, Oulu, Finland; Biocenter Oulu, University of Oulu, Oulu, Finland; Pediatric Gastroenterology and Metabolic Diseases, Pediatric Institute, Poznan University of Medical Sciences, Poznan, Poland; Medical Research Center (MRC), Oulu University Hospital, Oulu, Finland

## Abstract

Physical activity (PA) has a positive effect on risk factors related to cardiometabolic health yet amount of PA and time of onset is unclear. Therefore, we investigated the relationship of PA estimates and cardiometabolic risk factors in a large healthy population of an understudied age group of young adults using a standard gravity-based method on body adiposity and risk markers. In 856 (532 women, 324 men, 32–35 years) subjects we evaluated the association of PA and cardiometabolic risk factors in early adulthood. PA was measured using accelerometers for a period of two weeks. Step counts were divided into light (LPA), moderate (MPA), and vigorous (VPA) intensity classes. Income of the household was 63 446 ± 46 899€ and 57.5% had higher education. Total daily step numbers were 11962.5 ± 5163.2, LPA 5459.6 ± 2986.6, MPA 5932 ± 3404.6, and VPA 572.3 ± 668. Higher total PA volume was associated with lower weight, BMI, % body fat, smaller visceral fat area (VFA) and waist circumference, lower total cholesterol, LDL, and reflection coefficient of the pulse wave. LPA correlated with weight, BMI, waist circumference, total cholesterol, LDL, and central pulse pressure (cPP). Percent body fat (%BF), VFA, total cholesterol, LDL, reflection coefficient, heart minute index, and heart minute volume were significantly associated with MPA and VPA intensity PA volume. Lower PA in early adulthood correlates with increased cardiometabolic risk markers which should be translated into specific recommendations to thrive for a healthier lifestyle to delay and decrease their onset.

## Introduction

In the European Union (EU), 90% of all deaths are caused by non-communicable diseases and 40% by cardiovascular diseases (CVD) [1]. The European Heart Network has estimated that CVD causes costs over 200 billion euro per year [2]. In addition, the prevalence of obesity (BMI ≥30 kg/m) has more than doubled across the EU over the course of 35 years, affecting more than 20% of adults. By eliminating the main lifestyle risk factors for type 2 diabetes (T2D), 93% of these cases of 35–65-year-old adults could be preventable [1]. Prevention has the highest success rate if applied early with common and cheap countermeasures when tissues have not changed into a pathophysiological phenotype. Physical activity (PA) is cheap and scientifically proven benefits in changing blood pressure, high- and low-density lipoproteins and total cholesterol levels, triglycerides, inflammation, plasma glucose, and insulin levels [3–8].

PA is beneficial in all intensities but has the strongest effects with moderate to vigorous physical activity (MVPA) on cardiometabolic health [4, 6]. A prospective cohort (aged 18–75 years at baseline) with 15 years follow-up time found that 30 min of total PA per day was associated to a 50% lower mortality rate for all-cause and CVD mortality [9]. In a cross-sectional study, reallocating 30 min from sedentary behavior (SB) to MVPA had significant benefits on HDL cholesterol, triglycerides, waist circumference, plasma glucose, and insulin levels [10]. The recommended 150 min of MVPA/week was associated with 1% lower carotid-femoral pulse wave velocity corresponding to 1.8 years reduction in vascular age [8]. Tudor-Locke and colleagues found that normal weight subjects took more steps per day and spent more time in MPA and VPA than the overweight or obese subjects [11]. One hundred fifty minutes of MVPA per week was more beneficial to cardiometabolic health than 10 000 steps/day target, suggesting that the impact of steps on health effects could vary.

The development of a cardiometabolic risk profile starts early with a reduction in physical activity. We therefore wanted to investigate the relationship of PA estimates and cardiometabolic risk factors in a large healthy young population using a standard gravity (*g*) based method on body adiposity and blood-derived risk markers. We hypothesize that already at a young health stage adiposity and cardiovascular markers are inversely correlated with PA.

## Methods

**Figure 1.**
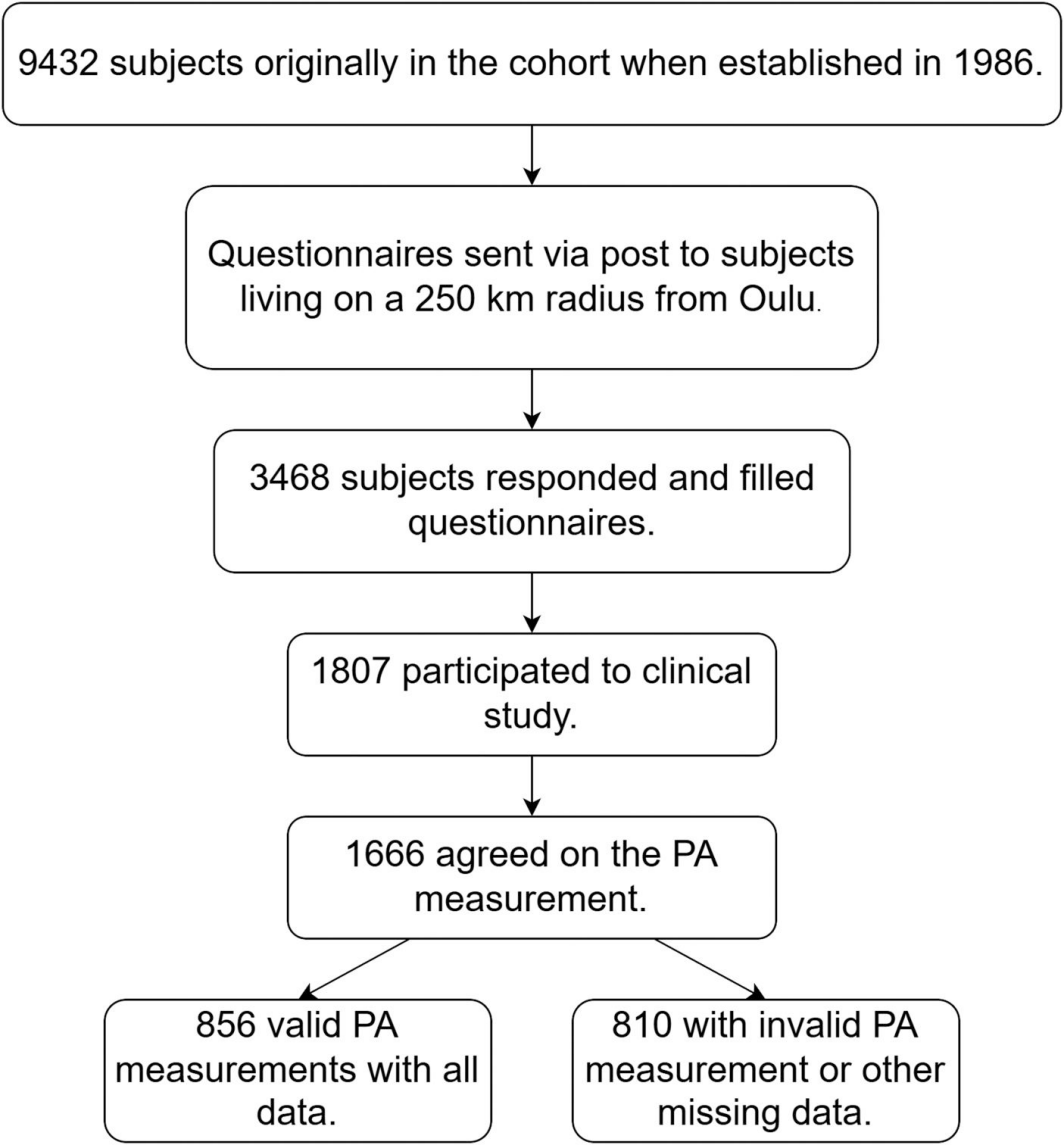
The flow chart of the study.

We measured healthy subjects that belonged to the Northern Finland Birth Cohort (NFBC1986) which were invited for a data collection during May 2019–December 2020 (Fig. 1) [12]. The original cohort consisted of 99% of born children between 1 July 1985 and 30 June 1986 in the two most Northern provinces in Finland. The participants living near the Oulu region were sent an invitation to participate in the data collection. A total of 3468 subjects filled in the broad postal questionnaires which included background (I), health (II), economy, work, and mental resources questionnaire (III), opinions and experiences questionnaires (IV). The data collection coincided with the COVID-19 pandemic, which required a reorganization of the data collection procedures. A total of 1807 subjects participated in the on-site measurements and 1666 agreed to the 2-week PA measurement of whom 856 (532 women and 324 men) completed the measurement. The acceptance criteria for the PA measurement were at least 7 days of valid wear-time and a filled PA diary (including working days, sleeping periods, and possible non-wear reasons). The wear-time was determined from the diaries filled by the participant. One valid day was 16 h or the wear-time from waking up to going to sleep. At least five working days and two weekend days were needed for a valid measurement period. Table 1 shows the population characteristics. The study was conducted according to the declaration of Helsinki, national law and was reviewed and approved by the Northern Ostrobothnia Hospital District Ethical Committee 108/2017 (15 January 2018). Informed consent was obtained from all subjects in the study.

**Table 1.** The characteristics of the study population (*n* = 856)

	Men (*n* = 324)		Women (*n* = 532)		All (*n* = 856)	
Income						
Total household (€/year, mean)	62 584 ± 26 609		64 013 ± 56 411		63 446 ± 46 899	
Education						
Upper-secondary education or lower-level education (%)	8.1		7.1		7.5	
Short cycle tertiary education (%)	41.8		30.4		34.8	
Higher education (%)	49.8		62.3		57.5	
Other (%)	0.3		0.2		0.2	
	Mean ± SD	Min.–Max.	Mean ± SD	Min.–Max.	Mean ± SD	Min.–Max.
Hemoglobin-A1c (mmol/mol)	33.5 ± 3.6	24–74	32.9 ± 4.3	25–107	33.1 ± 4.1	24–107
Alanine aminotransferase (U/l)	36.5 ± 25.3	9–189	19.9 ± 13.4	0–164	26.2 ± 20.4	0–189
Aspartate aminotransferase (U/l)	21.6 ± 13.3	0–145	15.9 ± 14.0	0–218	18 ± 14.0	0–218
Albumin (g/l)	41.9 ± 2.2	35–47	39.4 ± 2.8	26–46	40.4 ± 2.8	26–47
Glucose (mmol/l)	5.2 ± 0.8	42739	4.9 ± 0.8	3.8–21.2	5 ± 0.8	3.8–21.2
C-reactive protein (mg/l)	1.2 ± 2.5	0–34.7	1.8 ± 3.1	0–24.6	1.6 ± 2.9	0–34.7
Cholesterol (mmol/l)	4.8 ± 0.8	2.8–8.6	4.6 ± 0.8	2.4–8.6	4.6 ± 0.8	2.4–8.6
High density lipoprotein (mmol/l)	1.4 ± 0.3	0.7–2.8	1.6 ± 0.3	0.88–2.85	1.5 ± 0.3	0.72–2.85
Low density lipoprotein (mmol/l)	3.0 ± 0.7	1.3–5.7	2.5 ± 0.7	0.5–5.4	2.7 ± 0.8	0.5–5.7
Triglycerides (mmol/l)	1.0 ± 0.8	0.3–8.8	0.8 ± 0.4	0.18–3.81	0.9 ± 0.6	0.18–8.82
Height (cm)	178.7 ± 6.0	160.4–200.8	165.0 ± 5.7	148–180.4	170.2 ± 8.8	148–200.8
Weight (kg)	82.9 ± 13.2	51.2–143.2	69.8 ± 14.4	39.7–129.6	74.7 ± 15.3	39.7–143.2
Waist circumference (cm)	92.1 ± 10.3	68.5–138.5	84.6 ± 12.9	0–170.5	87.3 ± 12.6	0–170.5
Hip circumference (cm)	99.7 ± 7.00	80.5–132.5	99.1 ± 11.6	0–146.5	99.3 ± 10.1	0–146.5
BMI (body mass index)	25.95 ± 3.74	17.8–44	25.6 ± 4.97	15–45.9	25.7 ± 4.5	15–45.9
VFA (visceral fat area)	79.1 ± 43.4	5–301.3	107.6 ± 56.5	17.6–281.2	96.8 ± 53.7	5–301.3
Grip strength (kg)	52.3 ± 7.8	27–78	32.9 ± 5.5	4–60	40.2 ± 11.4	4–78
Total alcohol intake (g/day)	5.8 ± 9.5	0–65.3	3.0 ± 7.4	0–91.3	4.0 ± 8.6	0–91.3
Systolic blood pressure (mmHg)	125.8 ± 11.2	103.7–176	114.9 ± 9.8	91.7–155.3	119.0 ± 11.6	91.7–176
Diastolic blood pressure (mmHg)	80.6 ± 8.3	60.3–108	74.7 ± 8.6	53.3–106.3	76.9 ± 8.9	53.3–108
Pulse wave velocity (PWV)	5.74	5.0–7.4	5.36 ± 0.32	4.33–6.73	5.5 ± 0.4	4.33–7.4

Men (*n* = 324) and women (*n* = 532). Results are presented as mean ± SD.

Subjects wore the Sartorio Xelometer (Oulu, Finland) on their right hip for two consecutive weeks, advised only to remove it when showering, swimming, going to sauna, and sleeping. Sartorio Xelometer is a tri-axial accelerometer with a raw acceleration data output (*g*) with a 16 g range, 100 Hz sampling rate, and a battery life of 21 days of measurement [13, 14]. Measurement data were extracted using Sartorio v18 software and detection algorithms provided by the manufacturer were run on MATLAB R2019a for step counts and step intensities as 3D-acceleration vectors (*g*) [13]. The acceleration data was analysed as a whole, without setting any epochs. The step detection algorithm was based on the following variables: (1) A threshold value for the 3D acceleration. (2) The maximum value of the 3D acceleration peak. (3) The slope of the 3D acceleration peak. (4) The area of the 3D acceleration peak. (5) The time difference between consecutive 3D acceleration peaks. The algorithm was optimized with data from 35 participants and validated in a cohort of 19 normal weighted participants and 48 overweight participants [13, 14]. After detection of step numbers and their intensities (acceleration, *g*), step counts were divided into light, moderate, and vigorous according to corresponding acceleration in *g*-values and MET-values derived from previous measurements on a treadmill in a laboratory setting [13, 14]. The laboratory setting included male and female participants (*N* = 83) with varying body anthropometrics and age (21–74 years old). Steps were categorized into intensity classes as follows: Light (LPA) 1.037–1.67 g (<3 METs), moderate (MPA) 1.67–2.5 g (3–6 METs), and vigorous (VPA) 2.5–10 g (>6 METs). LPA, MPA, and VPA variables represent the daily step numbers within the category cut-offs. Sedentary behavior was not studied. Anthropometric measures, pulse wave analysis (Mobil-O-Graph, IEM GmbH, Aachen, Germany), blood pressure measurement, grip strength, and blood drawing were conducted by a registered study nurse following the best standard procedures. Biological samples were analysed daily in Nordlab (Nordlab, Oulu, Finland). Body mass index (BMI), percent body fat (%BF), and visceral fat area (VFA) were obtained from bioimpedance analysis, with InBody 720 device (Biospace, Co, Ltd, Seoul, Korea). Statistical analysis was performed using IBM SPSS Statistics 27. Normality of the data was tested using the Kolmogorov–Smirnov test. Then, Pearson correlations were used to assess associations between variables of interests and statistically significant correlations were chosen for additional analysis. Observed significant associations between PA and CVD risk variables were further examined using multivariable linear regression with alcohol consumption (g/day), smoking status (yes/no), and education level as covariates (model 1) (Table 2 and Supplementary Table S1). Adjusted and unadjusted *R*^2^-values were calculated to assess the fit of the model. Multicollinearity was assessed using the variance inflation factor (VIF) and values over 5 were considered to have severe multicollinearity. The level of statistical significance was set to 0.05. The analyses were conducted separately for the whole study population and for men and women separately to study whether there are sex differences in PA response with individual variables. Data are presented as mean ± SD.

**Table 2.** The PA variable characteristics of the population in a day

	Men (*n* = 324)		Women (*n* = 532)		All (*n* = 856)	
	Mean ± SD	Min.–Max.	Mean ± SD	Min.–Max.	Mean ± SD	Min.–Max.
Total steps	11946.6 ± 5441.2	2032.5–30953.2	11972.1 ± 4992.5	2197.2–26431.6	11962.5 ± 5163.2	2032.5–30953.2
LPA	5359.7 ± 2968.4	995.2–18339.9	5520.5 ± 2998.9	1031.4–22808.6	5459.6 ± 2986.6	995.2–22808.6
MPA	5983.8 ± 3574.6	566.1–20400.4	5900.9 ± 3301.6	553.4–19514.4	5932 ± 3404.6	553.4–20400.4
VPA	631.5 ± 703.6	10.5–5838.1	535.9 ± 643.1	11.2–5447.5	572.3 ± 668	10.5–5838.1

Mean step numbers, light physical activity (LPA) step numbers, moderate physical activity (MPA) step numbers, and vigorous physical activity (VPA) step numbers.

## Results

The results are based on the valid data on 856 healthy 32–35-year-old adults (Table 1). The mean combined income of the household was 63 446 ± 46 899€ and 57.5% had higher education degrees. The total mean step numbers were 11962.5 ± 5163.2 per day (Table 2). The proportion of LPA (1.037–1.67 g) category steps was 5459.6 ± 2986.6, MPA (1.67–2.5 g) was 5932 ± 3404.6, and VPA (2.5–10 g) was 572.3 ± 668. The observed step numbers were similar in both sexes. Men had 17.9% more VPA than women. The adjusted coefficients of determination ranged between 0.007 and 0.059 for statistically significant associations. Total step counts correlated with several anthropometric and adiposity-describing variables (Table 3). Higher total PA volume was associated with lower weight, BMI, %BF, smaller VFA and waist circumference, lower total cholesterol, LDL, reflection coefficient (ratio of the amplitudes of the two waves during one cardiac cycle), and a higher heart minute volume index.

**Table 3.** Multiple linear regression analysis results on total PA, light, moderate, and vigorous volumes (continuous scale) and cardiovascular risk factors

		Total steps				Light				Moderate				Vigorous			
		Beta	*P*	*R* ^2^	Adj. *R*^2^	Beta	*P*	*R* ^2^	Adj. *R*^2^	Beta	*P*	*R* ^2^	Adj. *R*^2^	Beta	*P*	*R* ^2^	Adj. *R*^2^
Weight	All	−0.147	.000	0.041	0.036	−0.080	.025	0.029	0.024	−0.121	.001	0.037	0.032	−0.044	.227	0.025	0.020
	Male	−0.202	.001	0.054	0.039	−0.105	.075	0.023	0.009	−0.196	.001	0.05	0.036	−0.108	.074	0.023	0.009
	Female	−0.113	.016	0.017	0.008	−0.054	.240	0.011	0.003	−0.093	.042	0.017	0.009	−0.063	.172	0.012	0.004
BMI	All	−0.136	.000	0.034	0.029	−0.072	.047	0.021	0.016	−0.126	.000	0.031	0.026	−0.093	.011	0.024	0.019
	Male	−0.171	.004	0.058	0.045	−0.102	.083	0.040	0.026	−0.163	.006	0.055	0.042	−0.082	.173	0.036	0.022
	Female	−0.121	.009	0.026	0.017	−0.058	.205	0.015	0.006	−0.111	.016	0.023	0.015	−0.099	.034	0.021	0.012
VFA	All	−0.139	.000	0.028	0.022	−0.049	.173	0.011	0.006	−0.134	.000	0.026	0.021	−0.170	.000	0.037	0.032
	Male	−0.165	.005	0.063	0.049	−0.058	.321	0.039	0.025	−0.180	.002	0.068	0.054	−0.140	.019	0.054	0.041
	Female	−0.144	.002	0.033	0.024	−0.066	.154	0.016	0.008	−0.125	.007	0.028	0.019	−0.164	.000	0.039	0.030
%BF	All	−0.121	.001	0.018	0.013	−0.032	.384	0.005	0.000	−0.118	.001	0.018	0.012	−0.194	.000	0.041	0.035
	Male	−0.145	.013	0.061	0.047	−0.041	.488	0.041	0.027	−0.166	.004	0.067	0.054	−0.128	.032	0.055	0.042
	Female	−0.165	.000	0.039	0.031	−0.076	.101	0.018	0.010	−0.138	.003	0.031	0.023	−0.213	.000	0.057	0.049
Waist circumference	All	−0.115	.003	0.023	0.018	−0.097	.007	0.038	0.033	−0.125	.000	0.044	0.039	−0.094	.010	0.037	0.032
	Male	−0.101	.117	0.017	0.001	−0.119	.042	0.048	0.035	−0.183	.002	0.068	0.054	−0.131	.028	0.051	0.037
	Female	−0.115	.019	0.019	0.009	−0.079	.086	0.020	0.012	−0.107	.020	0.025	0.017	−0.114	.014	0.027	0.019
Hip circumference	All	−0.032	.415	0.002	−0.004	−0.068	.058	0.015	0.009	−0.104	.004	0.021	0.016	−0.081	.027	0.016	0.011
	Male	−0.034	.594	0.006	−0.011	−0.104	.080	0.021	0.008	−0.171	.004	0.040	0.026	−0.092	.127	0.019	0.005
	Female	−0.033	.500	0.010	0.000	−0.058	.202	0.014	0.006	−0.082	.074	0.018	0.009	−0.082	.075	0.018	0.009
Cholesterol	All	−0.109	.002	0.026	0.021	−0.088	.014	0.022	0.017	−0.078	.031	0.020	0.015	−0.066	.070	0.018	0.013
	Male	−0.105	.075	0.030	0.016	−0.04	.504	0.020	0.006	−0.107	.069	0.030	0.016	−0.117	.053	0.032	0.018
	Female	−0.118	.010	0.021	0.013	−0.111	.015	0.020	0.012	−0.067	.146	0.012	0.004	−0.064	.161	0.012	0.003
LDL	All	−0.124	.001	0.028	0.023	−0.088	.015	0.020	0.015	−0.097	.007	0.022	0.017	−0.082	.023	0.019	0.014
	Male	−0.128	.031	0.028	0.014	−0.064	.283	0.016	0.002	−0.113	.056	0.024	0.010	−0.165	.006	0.038	0.024
	Female	−0.129	.005	0.023	0.015	−0.092	.043	0.015	0.007	−0.098	.032	0.016	0.008	−0.076	.100	0.012	0.004
Triglycerides	All	−0.066	.066	0.026	0.021	−0.040	.260	0.023	0.018	−0.055	.123	0.025	0.019	−0.051	.159	0.024	0.019
	Male	−0.040	.493	0.049	0.036	0.013	.826	0.048	0.034	−0.062	.286	0.052	0.038	−0.053	.372	0.051	0.037
	Female	−0.113	.013	0.017	0.009	−0.093	.042	0.013	0.005	−0.073	.109	0.010	0.002	−0.075	.104	0.010	0.002
cPP	All	−0.056	.136	0.030	0.025	−0.099	.009	0.024	0.018	−0.058	.125	0.017	0.011	−0.047	.216	0.016	0.010
	Male	−0.124	.042	0.056	0.042	−0.121	.047	0.056	0.041	−0.080	.191	0.048	0.033	−0.070	.261	0.046	0.031
	Female	−0.079	.105	0.009	0.000	−0.066	.170	0.007	−0.002	−0.048	.323	0.005	−0.004	−0.057	.242	0.006	−0.003
Reflection coefficient	All	−0.121	.001	0.018	0.013	−0.088	.020	0.012	0.006	−0.077	.043	0.010	0.004	−0.153	.000	0.027	0.021
	Male	−0.186	.003	0.044	0.029	−0.084	.178	0.016	0.001	−0.183	.003	0.042	0.027	−0.181	.004	0.040	0.025
	Female	−0.080	.099	0.008	−0.002	−0.098	.043	0.011	0.002	−0.006	.902	0.001	−0.008	−0.130	.008	0.018	0.009
Heart rate	All	−0.046	.227	0.005	−0.001	0.022	.568	0.003	−0.002	−0.061	.109	0.007	0.001	−0.131	.001	0.020	0.014
	Male	−0.005	.935	0.027	0.012	0.066	.288	0.032	0.017	−0.040	.512	0.029	0.014	−0.101	.109	0.037	0.022
	Female	−0.088	.068	0.011	0.002	−0.023	.639	0.004	−0.006	−0.085	.080	0.010	0.001	−0.131	.007	0.020	0.011
Heart minute volume index	All	0.088	.021	0.010	0.004	0.053	.167	0.005	−0.001	0.080	.036	0.008	0.002	0.047	.225	0.004	−0.002
	Male	0.041	.511	0.019	0.004	0.019	.760	0.018	0.003	0.041	.507	0.019	0.004	0.032	.619	0.018	0.003
	Female	0.117	.015	0.020	0.011	0.071	.140	0.012	0.003	0.101	.037	0.017	0.008	0.063	.196	0.011	0.001

Beta coefficients, statistical significance levels (*P*-values), and unadjusted and adjusted *R*^2^-values are presented for total population (*n* = 856), and men and women separately and adjusted for education, smoking, and alcohol intake.

For men, the higher total step count was associated after adjusting for cofounders with lower weight, BMI, %BF, smaller VFA, LDL cholesterol, cPP, and reflection coefficient. Light intensity PA volume correlated with waist circumference and lower cPP in model 1. In the multivariable regression model, moderate intensity PA volume was associated with lower weight, BMI, %BF, reflection coefficient and smaller VFA, waist and hip circumference. With vigorous PA intensity, %BF, LDL cholesterol, reflection coefficient, VFA, and waist circumference significantly correlated in model 1 (Table 3).

For women, higher total step count associated in model 1 with weight, BMI, %BF, total cholesterol, LDL cholesterol, triglycerides, VFA, waist circumference, and heart minute volume. In model 1, light intensity PA volume was correlated with total cholesterol, LDL cholesterol, triglycerides and reflection coefficient. Moderate PA was associated with weight, BMI, VFA, LDL cholesterol, waist circumference, %BF, and heart minute volume remained. With vigorous intensity PA volume, BMI, VFA, total cholesterol, LDL cholesterol, waist circumference, %BF, reflection coefficient, and HR stayed significant were associated in the model (Table 3).

## Discussion

In a large data collection in Northern Finland (latitude 65° North) the relationship between standard gravity-based PA variables and cardiometabolic risk factors were analysed. Step numbers as total and intensity-wise were similar between sexes with the exception of men having more VPA, which has been reported in Norway in 20–85 year-old subjects [15]. With increasing intensity level, we observed an increased number of associations: For men the associations with total step numbers included weight, BMI, %BF, VFA, LDL, central PP, and reflection coefficient. For women total cholesterol, triglycerides, waist circumference, and heart minute volume were associated but not central PP nor reflection coefficient.

We found significant associations mainly on body adiposity and lipid biomarkers. Increasing PA intensity associations had stronger correlation coefficient with variables such as abdominal adiposity, and percentage of body fat. Similar findings in a population with matching age-structure were reported in Brazil with BMI, waist circumference, VFA, and %BF [16]. In terms of PA intensities, benefits were already gained with LPA with additional effects with MPA and VPA [4, 6], especially regarding body adiposity [17–19]. A Canadian study with participants with a mean age of 41.5 ± 14.9 years found that the PA recommendations were associated with BMI, blood pressure, HDL and LDL, blood glucose, and insulin and beneficial effects towards BMI, blood pressure, HDL, total cholesterol, and insulin with lower PA volumes [5]. Chastin and colleagues reported similar findings with BMI and waist circumference, however they did not describe the relationship between PA and LDL or total cholesterol, but the authors found significant associations with glucose metabolism most likely due to the large age variation (21–64 years) [6]. Several studies described the association between PA and HDL, which we did not observed, but found an association between PA and LDL [18, 20]. Our subjects were active with nearly 12 000 daily steps with large proportions of steps in LPA and MPA. Step numbers have been investigated in multiple studies with a large age distribution [21, 22]. Hansen and colleagues studied 2183 subjects aged 57.0 ± 10.9 years and reported 8002 daily steps on average with lowest quartile median being 4651 steps/d and highest 11 467 steps/d [23]. A study with 16 741 elderly female subjects (aged 72 (62–101)) reported 5499 steps/d [24]. In middle-aged subjects (mean age of 49.7 and 45.2), two studies reported daily step numbers of 9146 and 10 733 [21, 25]. Our population was younger, explaining partly their higher step numbers. There is a lack of studies examining objectively measured PA and health parameters overall in subjects aged 20–40, making direct comparisons of our results to others challenging. Importantly, if low-force steps are not detected or censored by the accelerometer, it shifts the distribution to the left, indicating more sedentary numbers [22].

Our participants were in an overall healthy state which is demonstrated by the fact that we did not find any associations with glucose levels and PA. Only 20 subjects had impaired fasting glucose values (over 6.1 mmol/l), indicating that impaired glucose levels appear after changes in BMI and lipid levels. The results regarding PA and LDL could be influenced by differences in body fat distribution and adiposity, differences in the subjects’ diets, or the combination of these three [26]. Associations between PA variables and lipid markers were significant at lower intensity classes in women and waist circumference correlated significantly with all intensity classes in women, but in men only at moderate and vigorous intensities. PA was correlated with blood pressure, reflection coefficient, and heart minute volume across all three intensities. Yet, even at this relatively young age, we found significant associations between PA levels and cardiometabolic health. Weight gain from early adulthood to middle age has been associated with increases in CVDs, T2D, hypertension, and cancer [27]. The adverse health effects of insufficient PA accumulate as we age. These effects may start with increases in body adiposity and abnormal lipid metabolism, followed by impaired glucose metabolism and subsequent development of disease phenotypes.

Our study has several strengths and weaknesses. The strengths of this study include a population of subjects which were of same age, born, and lived in the same regions, which reduced cultural variation and variation to lifestyle, ethnicity, or education quality. The single-center study design reduced differences in the methods caused by multi-center settings. Methods for collecting cardiometabolic variables were well established, tested, and conducted by trained, registered nurses. We measured physical activity over at least 7 days with a validated accelerometer. Accelerometry-based devices on the market are not comparable in count definition, step detection thresholds, sampling frequencies, data processing, raw data availability [28–30], while older and newer models do not always produce comparable data [31]. A weakness in our study was that the sex distribution was tilted towards women because they answered the study call more often and followed the instructions of measurements more closely. In addition, the data collection was affected by the COVID19 pandemic and its regulations, prolonging the study period. During the COVID19 pandemic, PA of young adults and late adolescents reportedly decreased compared to pre-COVID levels in Finland but for elderly people it has been shown to even increase [32, 33]. Thus, the PA levels of this population might have been lower than in their pre-COVID period and this might have affected the results depending on the individual’s participation period in relation to start of the pandemic and the possible decrease in PA levels has not affected the cardiometabolic risk factors yet. The COVID19 pandemic could also be viewed as strength, since we managed to conduct the measurement and observe the significant associations with PA and cardiometabolic variables even though measured population’s habitual PA levels might not represent pre-COVID levels.

In conclusion, standard gravity-based objective physical activity was associated with better body anthropometrics, lipid metabolism, and cardiovascular variables in a healthy, relatively young cohort. Men and women responded to physical activity intensity differently, with women having more associations towards better lipid metabolism at lower intensity levels. These clear associations might predict a higher risk profile for cardiometabolic diseases, but it is currently unknown which weight they carry for the individual. Longitudinal studies would be required. More research is warranted to elucidate the associations and especially thresholds between objectively measured PA and obesity, diabetes, and other life-style induced diseases in young adulthood, since these problems are becoming more common at even younger ages and contribute to health status later in individual’s life.

## Supplementary Material

ckae199_Supplementary_Data

## Data Availability

NFBC data are available from the University of Oulu, Infrastructure for Population Studies. Permission to use the data can be applied for research purposes *via* an electronic material request portal. In the use of data, we follow the EU general data protection regulation (679/2016) and the Finnish Data Protection Act. The use of personal data is based on a cohort participant’s written informed consent in their latest follow-up study, which may cause limitations to its use. Please, contact the NFBC project center (NFBCprojectcenter(at)oulu.fi) and visit the cohort website (www.oulu.fi/nfbc) for more information. Key points In a large cohort of young individuals, objective physical activity using a standard gravity (*g*) based method was significantly associated with better body anthropometrics, lipid metabolism, and cardiovascular variables. Men and women responded to physical activity intensity differently, with women having more associations towards better lipid metabolism at lower intensity levels. These associations suggest a higher risk for cardiometabolic diseases, which should be translated into specific recommendations to thrive for a healthier lifestyle to prevent, delay, or decrease their onset.

## References

[1] Vandenberghe D , Albrecht J. The financial burden of non-communicable diseases in the European Union: a systematic review. Eur J Public Health 2020;30:833–9. 10.1093/eurpub/ckz073 31220862

[2] Timmis A , Vardas P, Townsend N et al European Society of Cardiology: cardiovascular disease statistics 2021: executive summary. Eur Heart J Qual Care Clin Outcomes 2022;8:377–82. 10.1093/eurheartj/ehab892 35488372

[3] LaMonte MJ , Lewis CE, Buchner DM et al Both light intensity and moderate-to-vigorous physical activity measured by accelerometry are favorably associated with cardiometabolic risk factors in older women: the Objective Physical Activity and Cardiovascular Health (OPACH) Study. J Am Heart Assoc 2017;6:e007064. 10.1161/JAHA.117.007064 PMC5721888 29042429

[4] Howard B , Winkler EAH, Sethi P et al Associations of low- and high-intensity light activity with cardiometabolic biomarkers. Med Sci Sports Exerc 2015;47:2093–101. 10.1249/MSS.0000000000000631 25668400

[5] Hajna S , Ross NA, Dasgupta K. Steps, moderate-to-vigorous physical activity, and cardiometabolic profiles. Prev Med 2018;107:69–74. 10.1016/j.ypmed.2017.11.007 29126915 PMC6625960

[6] Chastin SFM , Palarea-Albaladejo J, Dontje ML et al Combined effects of time spent in physical activity, sedentary behaviors and sleep on obesity and cardio-metabolic health markers: a novel compositional data analysis approach. PLoS One 2015;10:e0139984. 10.1371/journal.pone.0139984 26461112 PMC4604082

[7] Andersson C , Lyass A, Larson MG et al Physical activity measured by accelerometry and its associations with cardiac structure and vascular function in young and middle-aged adults. J Am Heart Assoc 2015;4:e001528. 10.1161/JAHA.114.001528 25792127 PMC4392434

[8] Metsämarttila E , Rodilla E, Jokelainen J et al Effect of physical activity on pulse wave velocity in elderly subjects with normal glucose, prediabetes or type 2 diabetes. Sci Rep 2018;8:8045. 10.1038/s41598-018-25755-4 29795274 PMC5966452

[9] Dohrn I-M , Welmer A-K, Hagströmer M. Accelerometry-assessed physical activity and sedentary time and associations with chronic disease and hospital visits—a prospective cohort study with 15 years follow-up. Int J Behav Nutr Phys Act 2019;16:125. 10.1186/s12966-019-0878-2 31818303 PMC6902520

[10] Buman MP , Winkler EAH, Kurka JM et al Reallocating time to sleep, sedentary behaviors, or active behaviors: associations with cardiovascular disease risk biomarkers, NHANES 2005–2006. Am J Epidemiol 2014;179:323–34. 10.1093/aje/kwt292 24318278

[11] Tudor-Locke C , Brashear MM, Johnson WD et al Accelerometer profiles of physical activity and inactivity in normal weight, overweight, and obese U.S. men and women. Int J Behav Nutr Phys Act 2010;7:60. 10.1186/1479-5868-7-60 20682057 PMC2924256

[12] Järvelin MR , Elliott P, Kleinschmidt I et al Ecological and individual predictors of birthweight in a northern Finland birth cohort 1986. Paediatr Perinat Epidemiol 1997;11:298–312. 10.1111/j.1365-3016.1997.tb00007.x 9246691

[13] Stenbäck V , Leppäluoto J, Leskelä N et al Step detection and energy expenditure at different speeds by three accelerometers in a controlled environment. Sci Rep 2021;11:20005. 10.1038/s41598-021-97299-z 34625578 PMC8501125

[14] Stenbäck V , Leppäluoto J, Juustila R et al Step Detection Accuracy and Energy Expenditure Estimation at Different Speeds by Three Accelerometers in a Controlled Environment in Overweight/Obese Subjects. JCM 2022;11:3267. https://doi.org/10.3390/jcm11123267 35743338 PMC9224826

[15] Hansen BH , Kolle E, Dyrstad SM et al Accelerometer-determined physical activity in adults and older people. Med Sci Sports Exerc 2012;44:266–72. 10.1249/MSS.0b013e31822cb354 21796052

[16] Silva BGCD , Silva ICMD, Ekelund U et al Associations of physical activity and sedentary time with body composition in Brazilian young adults. Sci Rep 2019;9:5444. 10.1038/s41598-019-41935-2 30931983 PMC6443682

[17] Mac Ananey O , McLoughlin B, Leonard A et al Inverse relationship between physical activity, adiposity, and arterial stiffness in healthy middle-aged subjects. J Phys Act Health 2015;12:1576–81. 10.1123/jpah.2014-0395 25742623

[18] O'Donovan G , Hillsdon M, Ukoumunne OC et al Objectively measured physical activity, cardiorespiratory fitness and cardiometabolic risk factors in the Health Survey for England. Prev Med 2013;57:201–5. 10.1016/j.ypmed.2013.05.022 23732244

[19] Tudor-Locke C , Schuna JM, Han HO et al Step-based physical activity metrics and cardiometabolic risk: NHANES 2005–2006. Med Sci Sports Exerc 2017;49:283–91. 10.1249/MSS.0000000000001100 27669450 PMC5412514

[20] Atienza AA , Moser RP, Perna F et al Self-reported and objectively measured activity related to biomarkers using NHANES. Med Sci Sports Exerc 2011;43:815–21. 10.1249/MSS.0b013e3181fdfc32 20962693

[21] Paluch AE , Gabriel KP, Fulton JE et al Steps per day and all-cause mortality in middle-aged adults in the coronary artery risk development in young adults study. JAMA Netw Open 2021;4:e2124516. 10.1001/jamanetworkopen.2021.24516 34477847 PMC8417757

[22] Tudor-Locke C , Johnson WD, Katzmarzyk PT. Accelerometer-determined steps per day in US children and youth. Med Sci Sports Exerc 2010;42:2244–50. 10.1249/MSS.0b013e318199885c 20421837

[23] Hansen BH , Dalene KE, Ekelund U et al Step by step: association of device-measured daily steps with all-cause mortality – a prospective cohort study. Scand J Med Sci Sports 2020;30:1705–11. 10.1111/sms.13726 32427398 PMC7496562

[24] Lee I-M , Shiroma EJ, Kamada M et al Association of step volume and intensity with all-cause mortality in older women. JAMA Intern Med 2019;179:1105–12. 10.1001/jamainternmed.2019.0899 31141585 PMC6547157

[25] Ponsonby A-L , Sun C, Ukoumunne OC et al Objectively measured physical activity and the subsequent risk of incident dysglycemia: the Australian Diabetes, Obesity and Lifestyle Study (AusDiab). Diabetes Care 2011;34:1497–502. 10.2337/dc10-2386 21562319 PMC3120195

[26] Albarrati AM , Alghamdi MSM, Nazer RI et al Effectiveness of low to moderate physical exercise training on the level of low-density lipoproteins: a systematic review. Biomed Res Int 2018;2018:5982980. 10.1155/2018/5982980 30515408 PMC6236809

[27] Zheng Y , Manson JE, Yuan C et al Associations of weight gain from early to middle adulthood with major health outcomes later in life. JAMA 2017;318:255–69. 10.1001/jama.2017.7092 28719691 PMC5817436

[28] Fokkema T , Kooiman TJM, Krijnen WP et al Reliability and validity of ten consumer activity trackers depend on walking speed. Med Sci Sports Exerc 2017;49:793–800. 10.1249/MSS.0000000000001146 28319983

[29] John D , Morton A, Arguello D et al “What is a step?” Differences in how a step is detected among three popular activity monitors that have impacted physical activity research. Sensors (Switzerland) 2018;18:1206. 10.3390/s18041206 PMC5948774 29662048

[30] Leinonen A-M , Ahola R, Kulmala J et al Measuring physical activity in free-living conditions – comparison of three accelerometry-based methods. Front Physiol 2016;7:681. 10.3389/fphys.2016.00681 28119626 PMC5222829

[31] Smith MP , Horsch A, Standl M et al Uni- and triaxial accelerometric signals agree during daily routine, but show differences between sports. Sci Rep 2018;8:15055. 10.1038/s41598-018-33288-z 30305651 PMC6180043

[32] Ng K , Koski P, Lyyra N et al Finnish late adolescents’ physical activity during COVID-19 spring 2020 lockdown. BMC Public Health 2021;21:2197. 10.1186/s12889-021-12263-w 34852807 PMC8635322

[33] Lindeman K , Karavirta L, Eronen J et al Physical activity changes from before to during the first wave of the COVID-19 pandemic among community-dwelling older adults in Finland. J Aging Phys Act 2024;32:198–206. 10.1123/japa.2022-0281 38016452

