# Supplementary material for: Associations of step accelerations and cardiometabolic risk markers in early adulthood
Source: Eur J Public Health. 2024 Dec 4;35(1):128–33. doi: 10.1093/eurpub/ckae199 (PMC11832146; doi:10.1093/eurpub/ckae199)
Supplement: ckae199_Supplementary_Data [file ckae199_supplementary_data.docx]

Supplementary Table 1. Variables examined to assess the associations between physical activity and cardiometabolic risk factors.

| **Anthropometry** | **Blood markers** | **Cardiovascular variables** |
| --- | --- | --- |
| Height (cm) | Hemoglobin-A1c (mmol/mol) | Systolic blood pressure (mmHg) |
| Weight (kg) | Alkaline phosphatase (U/l) | Diastolic blood pressure (mmHg) |
| Waist circumference (cm) | Alanine aminotransferase (U/l) | Peripheral pulse pressure |
| Hip circumference (cm) | Aspartate aminotransferase (U/l) | Heart rate |
| BMI | Albumin (g/l) | Central pulse pressure |
| Visceral fat area | Amylase (U/l) | Central systolic blood pressure (mmHg) |
| Percentage body fat | Glutamyl transferase (U/l) | Central diastolic blood pressure (mmHg) |
|  | Glucose (mmol/l) | Cardiac stroke volume |
|  | Creatinine (umol/l) | Cardiac output |
|  | Urate (nl/l) | Pulse wave velocity |
|  | C-reactive protein (mg/l) | TVR |
|  | Cholesterol (mmol/l) | Augmentation index (Aix) |
|  | Cholesterol, HDL (mmol/l) | Reflection coefficient |
|  | Cholesterol, LDL (mmol/l) |  |
|  | Triglycerides (mmol/l) |  |
